# Supplementary material for: siRNA for REST ameliorates symptoms in ALS mice and serum REST predicts disease prognosis and survival in ALS patients
Source: Mol Ther. 2025 Oct 16;34(1):367–79. doi: 10.1016/j.ymthe.2025.10.039 (PMC12925782; doi:10.1016/j.ymthe.2025.10.039)
Supplement: Document S1. Figures S1–S4 [file mmc1.pdf]

YMTHE, Volume *34*

Supplemental Information

siRNA for REST ameliorates symptoms in ALS mice and serum REST predicts disease prognosis and survival in ALS patients

Natascia Guida, Valeria Valsecchi, Serenella Anzilotti, Raffaele Dubbioso, Ornella Cuomo, Silvia Ruggiero, Gianmaria Senerchia, Valentina Virginia Iuzzolino, Xhesika Kolici, Nunzia De Iesu, Giuseppe Pignataro, Lucio Annunziato, and Luigi Formisano

Supplemental Information

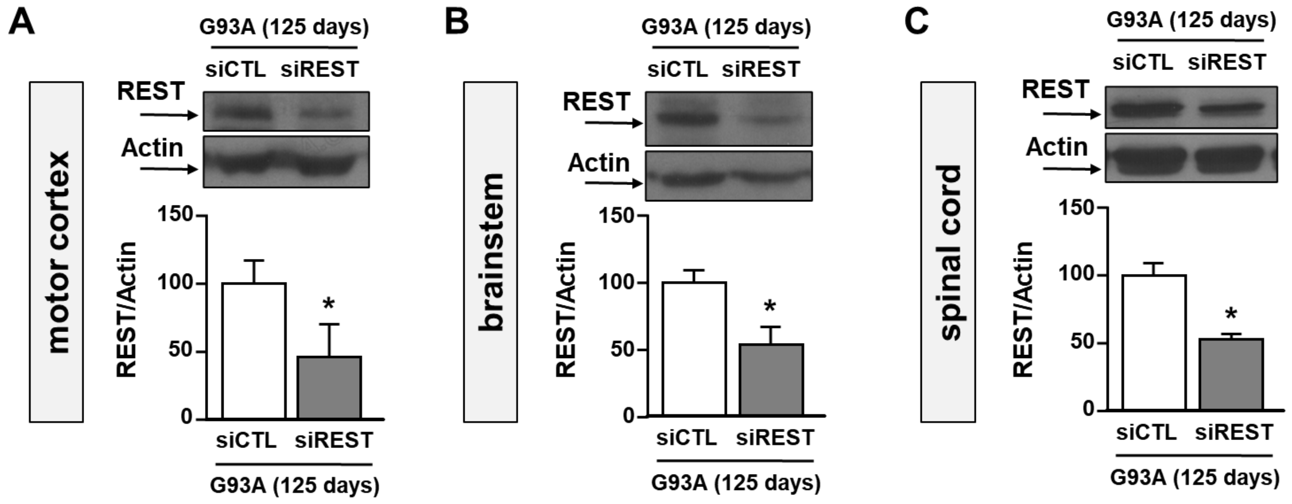

Figure S1

Effect of siREST icv injections on REST protein expression in motor cortex, brainstem and spinal cords of SOD1-G93A mice at 125 days of age. (A-C) Representative western blot and quantification of REST protein levels in motor cortex, brainstem and spinal cords of WT mice treated with siCTL or siREST and sacrificed at 125 days of age after 4 weeks of treatment (N=3). *P < 0.05 vs siCTL by student's t test.

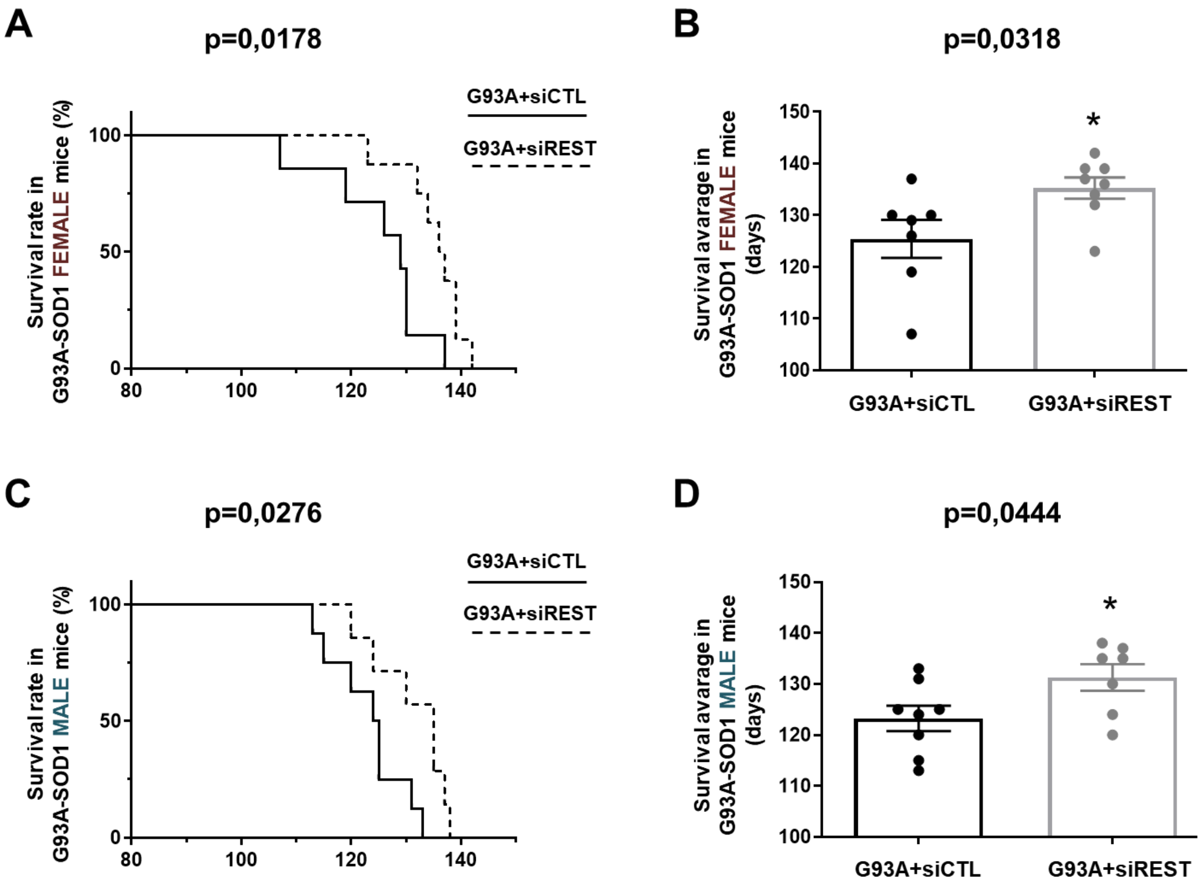

Figure S2

Sex-stratified survival analysis in G93A mice treated with siCTL or siREST. Kaplan-Meier survival analysis, expressed as a percentage (A, C) or in days (B, D) of G93A+siCTL (continuous line and black dots) and G93A+siREST (dashed line and grey dots) female mice (A, B) and male mice (C, D). *P < 0.05 by log-rank statistical test for (A, C) and *P < 0.05 by student's t test for (B, D).

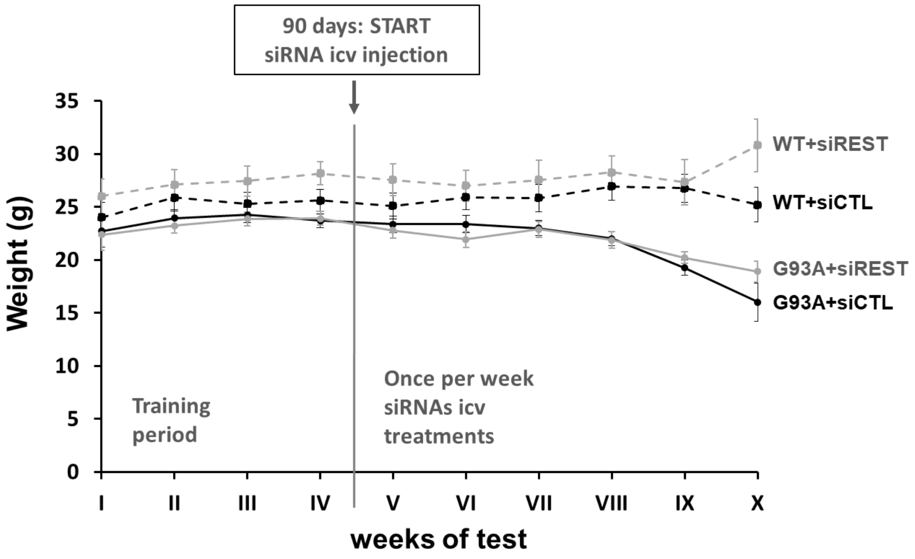

Figure S3

Effect of siREST treatment on body weight of ALS mice. Growth curves of: WT+siCTL (black dashed line; N=13) WT+siREST (gray dashed line; N= 14), G93A+siCTL (black continuous line; N= 28), G93A+siREST (gray continuous line; N= 34) mice. No significant differences by two-way ANOVA analysis, followed by Bonferroni post hoc test.

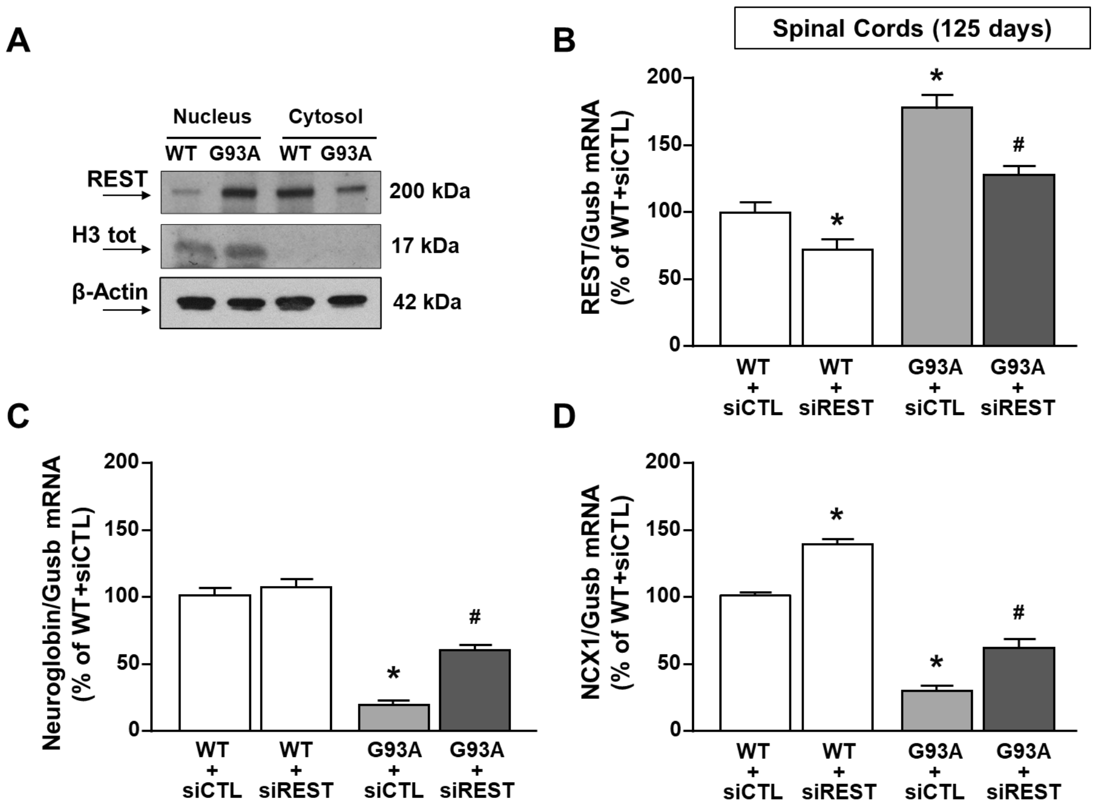

Figure S4

Nuclear and cytosolic REST protein expression and effect of siREST treatment on target genes expression in spinal cords of WT and G93A mice at 125 days of age. (A) Representative western blot of REST protein levels in nuclear and cytoplasmic differentiated lysates from spinal cords of WT and G93A mice at 125 days. Anti-histone H3 (H3) was used to verify the purity of the cytoplasmic and nuclear fractions (N = 3). (B-D) RT-PCR analysis showing REST, Neuroglobin and NCX1 gene expression in spinal cords from: WT+siCTL, WT+siREST, G93A+siCTL, G93A+siREST mice (N=5). *P < 0.05 vs WT+siCTL; #P < 0.05 vs G93A+siCTL by one-way ANOVA analysis, followed by Bonferroni post hoc test.
