# Supplementary material for: siRNA for REST ameliorates symptoms in ALS mice and serum REST predicts disease prognosis and survival in ALS patients
Source: Mol Ther. 2025 Oct 16;34(1):367–79. doi: 10.1016/j.ymthe.2025.10.039 (PMC12925782; doi:10.1016/j.ymthe.2025.10.039)
Supplement: Document S2. Article plus supplemental information [file mmc2.pdf]

Molecular Therapy
Original Article

siRNA for REST ameliorates symptoms in ALS mice and serum REST predicts disease prognosis and survival in ALS patients

Natascia Guida,[1] Valeria Valsecchi,[1,6] Serenella Anzilotti,[2,6] Raffaele Dubbioso,[3] Ornella Cuomo,[1] Silvia Ruggiero,[1] Gianmaria Senerchia,[3] Valentina Virginia Iuzzolino,[3] Xhesika Kolici,[1,4] Nunzia De Iesu,[5] Giuseppe Pignataro,[1] Lucio Annunziato,[5] and Luigi Formisano[1]

[1]Division of Pharmacology, Department of Neurosciences, Reproductive Sciences and Odontostomatology, University of Naples Federico II, 80131 Naples, Italy; [2]Department of Human Sciences and Quality of Life Promotion, San Raffaele University, 00166 Rome, Italy; [3]Clinical Neurophysiology Unit, Department of Neurosciences, Reproductive Sciences and Odontostomatology, University of Naples Federico II, 80131 Naples, Italy; [4]School of Advanced Studies, Center for Neuroscience, University of Camerino, Camerino, Italy; [5]IRCCS SYNLAB SDN, Via Galileo Ferraris 144, 80146 Naples, Italy

Restrictive element-1 silencing transcription factor (REST) is a key repressor of neuronal genes in stem cells and neuronal progenitor cells and its aberrant accumulation has been implicated in the pathophysiology of neurological disorders, such as Huntington's disease, epilepsy, and stroke. Herein, we investigated the role of REST in amyotrophic lateral sclerosis (ALS) pathophysiology and its potential as blood-based predictor of disease prognosis and survival in ALS patients. Intriguingly, REST protein levels were significantly increased in motor cortex, brainstem and spinal cord of superoxide dismutase 1 (SOD1)-G93A mice compared with wild-type mice, both during early and late symptomatic phases of the disease. Notably, intracerebroventricular injections of a siRNA against REST (siREST), mitigated motor neuron loss, counteracted the formation of SOD1 aggregates, and reduced astrogliosis, thus improving behavioral performance and extending the survival of SOD1-G93A mice. Interestingly, ELISA assay showed that serum REST levels were significantly elevated in ALS patients compared with healthy subjects; furthermore, the higher serum REST levels have been found in patients with shorter tracheostomy-free survival. Collectively, we demonstrated that preventing REST increase in brain areas involved in ALS disorder extended the survival of SOD1-G93A mice and showed that serum REST may represent a possible prognostic biomarker in ALS patients.

INTRODUCTION

The pathophysiological mechanisms underlying amyotrophic lateral sclerosis (ALS) are highly heterogeneous, involving complex interactions between genetic and environmental factors. A variety of pathophysiological mechanisms have been described, which include: glutamate excitotoxicity, free radical-mediated oxidative stress, neuroinflammation, and misfolded protein aggregates formation.[1] Most cases of ALS are sporadic and lack of any apparent genetic linkage, but approximately 10% of cases are familial (fALS), resulting from inherited genetic mutations. Of these familial cases, ∼20% have been attributed to mutations in a gene encoding for the ubiquitous cytoplasmic copper/zinc superoxide dismutase 1 (SOD1),[2] and overexpression of the human mutant SOD1-G93A in mouse models invariably results in motor neuron (MN) loss, muscle wasting, and hindlimb paralysis.[3]

Notably, we previously demonstrated that methylmercury (MeHg) hastens motor neuronal death in SOD1-G93A cells via the upregulation of the RE1-silencing transcription factor (REST), both at mRNA and protein levels.[4] The master neuronal transcriptional factor REST has a fundamental role in neuronal differentiation, structural remodeling, and plasticity,[5] and contributes to neuronal homeostasis in postnatal neurons.[6] In neurons REST is normally quiescent, meaning that it is at low levels and not actively repressing genes.[7] However, in neurological disorders, such as seizures,[8] stroke,[9,10] and Huntington's disease,[11] it plays a neurodetrimental role due to its accumulation in the nuclei of the neurons, where it represses genes involved in neuroprotection, such as AMPA receptor GluR2 subunit (GR2),[12] Na^+/Ca^{2+} exchanger 1 (NCX1),[10] and brain-derived neurotrophic factor (BDNF).[11]

Another important consideration is that REST can be detected and measured in blood, thus representing a possible biomarker in neurodegenerative diseases. Recently published studies demonstrated that REST plasma levels, including a free-floating

Received 12 May 2025; accepted 14 October 2025;
https://doi.org/10.1016/j.ymthe.2025.10.039.

[6]These authors contributed equally

Correspondence: Lucio Annunziato, IRCCS SYNLAB SDN, Via Galileo Ferraris 144, 80146 Naples, Italy.
E-mail: lannunzi@unina.it
Correspondence: Luigi Formisano, Division of Pharmacology, Department of Neurosciences, Reproductive Sciences and Odontostomatology, University of Naples Federico II, 80131 Naples, Italy.
E-mail: luigi.formisano@unina.it

Published by Elsevier Inc. on behalf of The American Society of Gene and Cell Therapy.
This is an open access article under the CC BY license (http://creativecommons.org/licenses/by/4.0/).

circulating REST and an exosome-derived amount, could be quantified in human blood samples by enzyme-linked immunosorbent assay (ELISA) assay.[13,14] In the present study we investigated whether the transcriptional factor REST is involved in ALS pathophysiology and its potential role as a blood-based biomarker of disease in ALS patients. To this aim we: (1) measured REST protein levels in motor cortex, brainstem, and spinal cord both during the early and late symptomatic phases of the disease in SOD1-G93A mice; (2) determined the effect of REST knockdown on MN survival and on astroglia and microglia activation in the spinal cord of SOD1-G93A mice; (3) analyzed the effect of REST knockdown on the formation of aggregates containing misfolded SOD1 in the spinal cord of SOD1-G93A mice; (4) evaluated whether the intracerebroventricular administration of short interfering RNA (siRNA) for REST ameliorated motor symptoms and prolonged survival rate in SOD1-G93A mice; (5) verified whether REST protein levels increased in the nuclear compartment of neurons from the spinal cord of SOD1-G93A mice and whether its levels affected the mRNA expression of ALS-related REST target genes; (5) finally, we measured REST plasma in ALS patients to verify whether it could be a diagnostic or prognostic biomarker.

RESULTS

REST protein levels increased in motor cortex, brainstem, and spinal cord both during the early and late symptomatic phases of the disease in SOD1-G93A mice

We measured REST protein expression in motor cortex, brainstem, and spinal cord of mice carrying the mutation G93A in the SOD1 gene (SOD1-G93A mice) at age 60, 105, and 125 days corresponding to the asymptomatic phase, the early symptomatic phase, and the late symptomatic phase of the disease, respectively. Western blotting experiments showed a significant increase of REST expression in motor cortex (Figure 1A), brainstem (Figure 1B), and spinal cord (Figure 1C) at 105 and 125 days, but not at age 60 days in ALS mice. Western blotting results were confirmed by confocal immunofluorescence analysis. Indeed, REST immunosignal was strongly increased in SMI32-positive cells, a specific marker for neurofilament protein, in the ventral horns of the lumbar spinal cord of SOD1-G93A mice at age 125 days compared with aged-matched wild-type (WT) mice (Figure 1D).

REST knockdown reduced MN loss, limited the formation of misfolded SOD1 aggregates, and prevented astroglia and microglia activation in the spinal cord of SOD1-G93A mice at age 125 days

To assess whether REST upregulation has a determinant role in the pathogenic mechanisms leading to ALS, we studied the effects of REST knockdown in SOD1-G93A mice. Specifically, a siRNA for REST (siREST) was intracerebroventricularly (i.c.v.) injected once a week, for 4 consecutive weeks, starting from age 90 days, a time that corresponds to 2 weeks before the occurrence of initial symptoms of the disease. Remarkably, REST protein levels were found to be significantly reduced in the motor cortex, brainstem, and spinal cord of SOD1-G93A mice at age 125 days, corresponding to 1 week after the last siREST i.c.v. injection, thus confirming the efficacy of siREST to prevent the increase of the transcription factor over time in all brain areas affected by the disorder (Figure S1).

More interestingly, REST knockdown prevented the reduction of the number of MNs in ALS mice, detected by Nissl staining in the ventral horns of the spinal cord at age 125 days. Furthermore, siREST administration did not alter the number of MNs in spinal cords of WT mice, which already displayed very low levels of the transcription factor (Figure 2A).

It is noteworthy that the enhanced formation of intracellular misfolded SOD1 is a well-characterized pathological feature of ALS.[15,16] To verify whether to limit the increase of REST could prevent the formation of aggregates containing misfolded SOD1, we performed an immunoprecipitation (IP) with B8H10, a monoclonal antibody that recognizes epitopes within exon 3, which are exposed only on misfolded or denaturated SOD1.[17] Interestingly, siREST treatment clearly reduced the accumulation of misfolded SOD1 in the spinal cord of SOD1-G93A mice at age 125 days (Figure 2B).

Next, it has been evaluated whether preventing REST accumulation in brain areas affected by ALS there was a reduction of astrogliosis and microgliosis, two well-characterized pathological hallmarks of ALS disease.[18,19] To this aim, the immunostaining of glial fibrillary acidic protein (GFAP) and ionized calcium-binding adapter molecule (IBA1), two markers of astrocytes and microglia, respectively, were evaluated in the ventral horns of the lumbar spinal cord of WT and late symptomatic SOD1-G93A mice. Remarkably, siREST treatment reduced astrogliosis and microgliosis in SOD1-G93A mice (Figures 3A, 3B, 3D, 3H, and 3L) compared with siCTL-treated SOD1-G93A mice.

i.c.v. administration of siRNA for REST ameliorated motor symptoms and prolonged survival rate in SOD1-G93A mice

To study the effect of REST knockdown on motor performance of ALS mice, behavioral tests were performed once per week on siCTL- or siREST-treated SOD1-G93A mice. In particular, starting 3 days after the first i.c.v. injection, the animals were subjected to rotarod and paw grip endurance (Grip) tests, the most adequate behavioral tests to analyze motor performance in ALS mice.[20]

Grip test analysis, an index of hindlimb muscle strength, measured in seconds, showed that SOD1-G93A mice treated with siREST displayed a mitigation in the impairment in motor performance compared with SOD1-G93A mice treated with siCTL. Notably, siREST in WT groups did not alter the Grip performance (Figure 4A). In particular, a significantly higher score for SOD1-G93A + siREST was observed at weeks 6 (SOD1-G93A + siCTL: 72.9 ± 3.7 s vs.

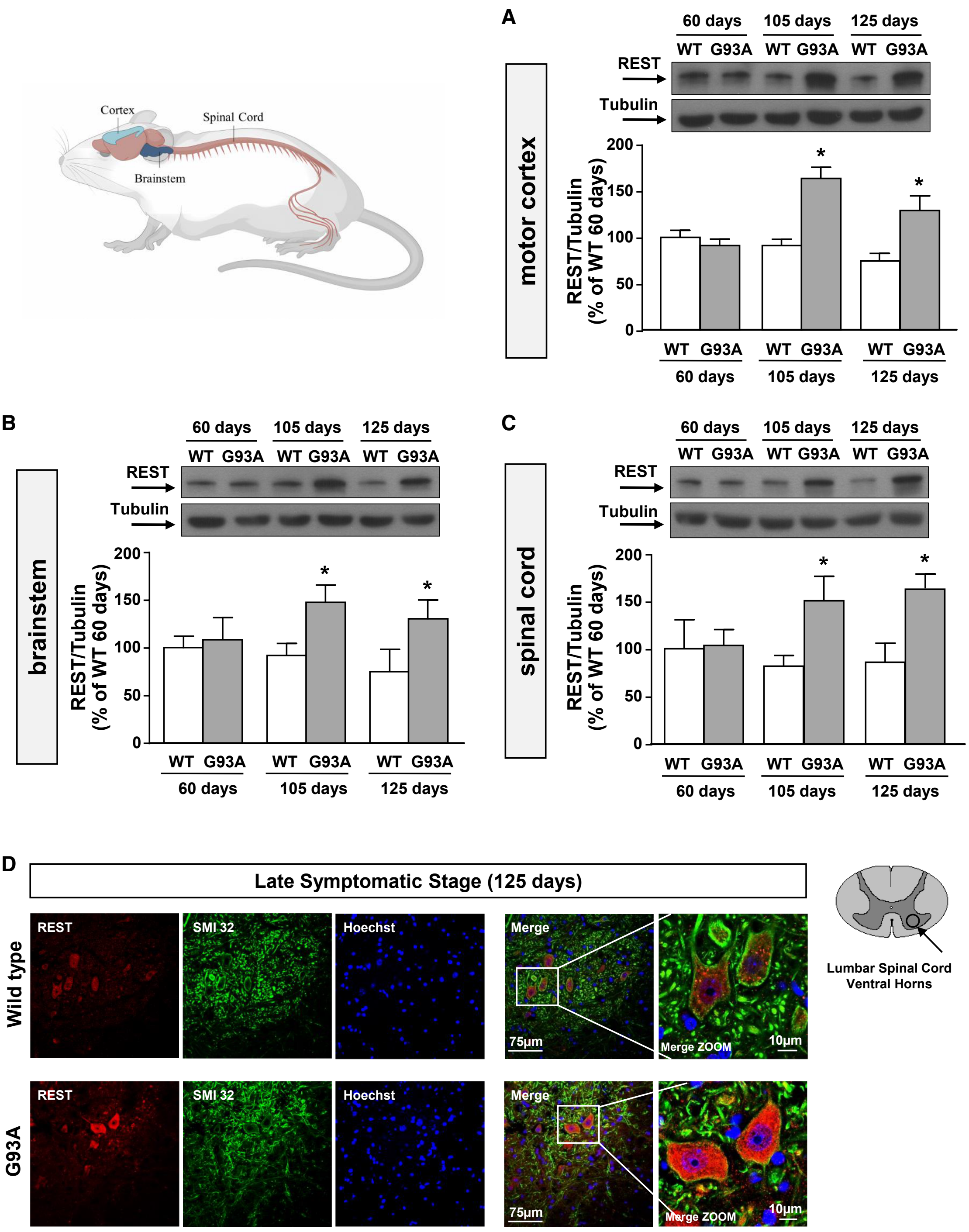

Figure 1. Characterization of REST protein expression in motor cortex, brainstem, and spinal cord of G93A mice, compared with WT mice, at early symptomatic phase or late phase of the disease

Representative western blot and quantification of protein extracts from (A) motor cortex, (B) brainstem, and (C) spinal cords in wild-type (WT) mice and SOD1-G93A (G93A) mice at age 60, 105, and 125 days ($n = 5$). $^*p < 0.05$ vs. aged-matched WT by two-way ANOVA analysis, followed by Bonferroni post hoc test. (D) Representative immunofluorescence images of REST and SMI32 colocalization in ventral horns of lumbar spinal cord sections from WT and G93A at 125 days ($n = 5$). Scale bars, 75 μm.

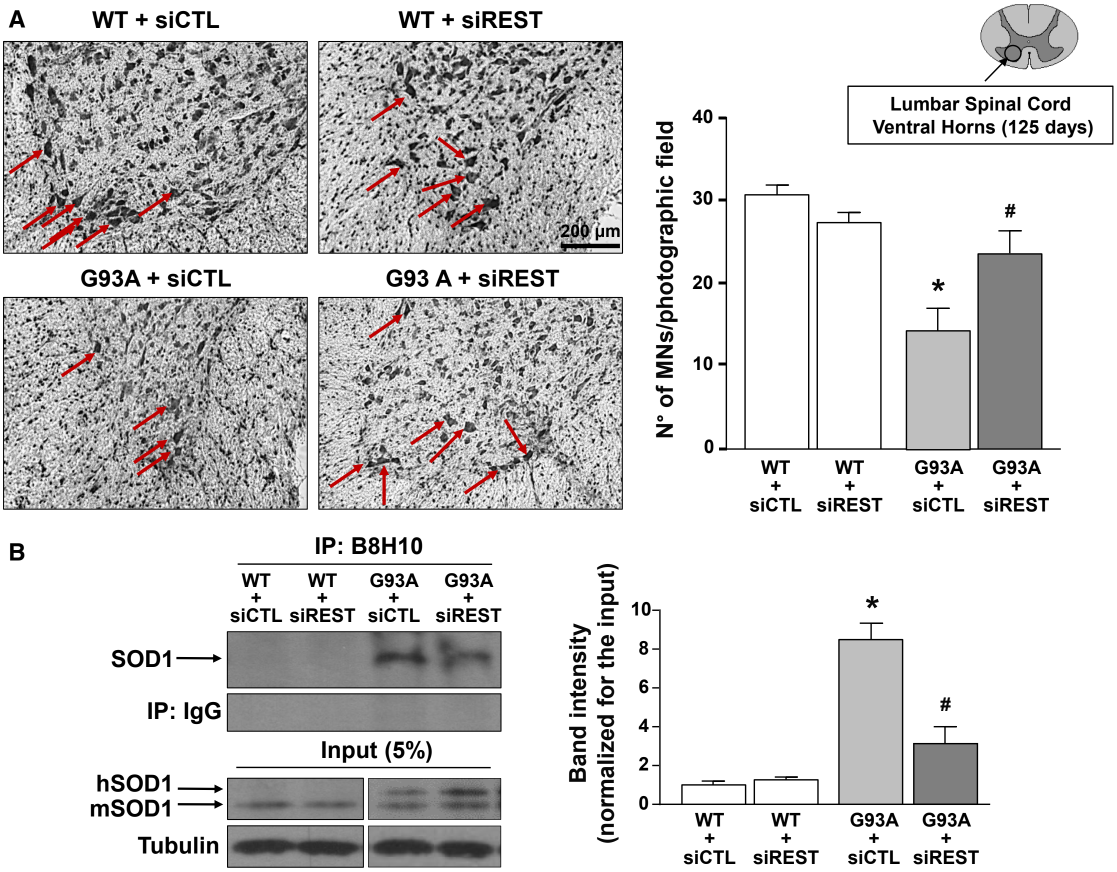

Figure 2. Effect of siRNA against REST on MN survival and formation of misfolded SOD1 aggregates in fully symptomatic G93A mice compared with age-matched WT mice

(A) (Left) Representative images of Nissl staining in lumbar spinal cords of WT and G93A (125 days) mice treated with siCTL or siREST. Scale bar, 200 μm. (Right) Cell counting analysis of MNs. MNs are expressed as number of MNs per photographic field in lumbar spinal cord ventral horn of: WT + siCTL, WT + siREST, G93A + siCTL, G93A + siREST mice ($n = 4$). *$p < 0.05$ vs. WT groups; #$p < 0.05$ vs. G93A + siCTL by one-way ANOVA analysis, followed by Bonferroni post hoc test. (B) Representative images (left) and quantification (right) of co-immunoprecipitation experiments performed in protein extracts of spinal cords from: WT + siCTL, WT + siREST, G93A + siCTL, G93A + siREST mice at 125 days. Mouse monoclonal antibody (B8H10), specific for misfolded forms of SOD1 has been used for immunoprecipitation. The 5% pre-immunoprecipitated protein lysates have been run as input. The antibody against the WT SOD1 was used for the immunoblotting. For quantification, the optical density (OD) of the bands of co-immunoprecipitation was normalized for the input OD. The WT + siCTL group was expressed as 1 and used for comparison with the other groups ($n = 3$). *$p < 0.05$ vs. WT + siCTL; #$p < 0.05$ vs. G93A + siCTL by two-way ANOVA analysis, followed by Bonferroni post hoc test.

SOD1-G93A + siREST: 83.6 + 2.5 s), 7 (SOD1-G93A + siCTL: 60.5 ± 3.8 s vs. SOD1-G93A + siREST: 73.6 + 4.1 s), and 8 (SOD1-G93A + siCTL: 31.3 ± 5.0 s vs. SOD1-G93A + siREST: 57.4 + 6.4 s) compared with the siCTL-treated ALS group (Figure 4B). Furthermore, motor coordination of SOD1-G93A mice treated with siCTL or siREST was assessed using the rotarod test. As expected, the treatment with siREST mitigated the decline in rotarod performance in SOD1-G93A mice. Moreover, WT mice were able to maintain balance for the duration of the test and siREST treatment did not alter their performance (Figure 4C). Specifically, a significant amelioration of the rotarod test was registered at weeks 7 (SOD1-G93A + siCTL: 116.0 ± 8.4 s vs. SOD1-G93A + siREST: 143.5 + 9.6 s), 8 (SOD1-G93A + siCTL: 79.1 ± 10.5 s vs. SOD1-G93A + siREST: 125.9 + 14.6 s), and 9 of the test (SOD1-G93A + siCTL: 34.4 ± 10.0 s vs. SOD1-G93A + siREST: 79.2 + 17.5 s) (Figure 4D).

Importantly, the knockdown for REST extended the lifespan of ALS mice. Indeed, the average lifespan of SOD1-G93A mice treated with siREST was significantly longer (133.2 ± 1.7 days) compared with ALS mice treated with siCTL (124.3 ± 2.1 days) (Figures 4E and 4F).

Specifically, siREST-treated female mice survived for 135.3 ± 2.1 days, approximatively 4 days longer compared with siREST-treated male mice (131.3 ± 3.6 days). However, although stratified by sex, the differences in the survival rate among male and female siREST-treated mice did not result statistically significant (Figure S2).

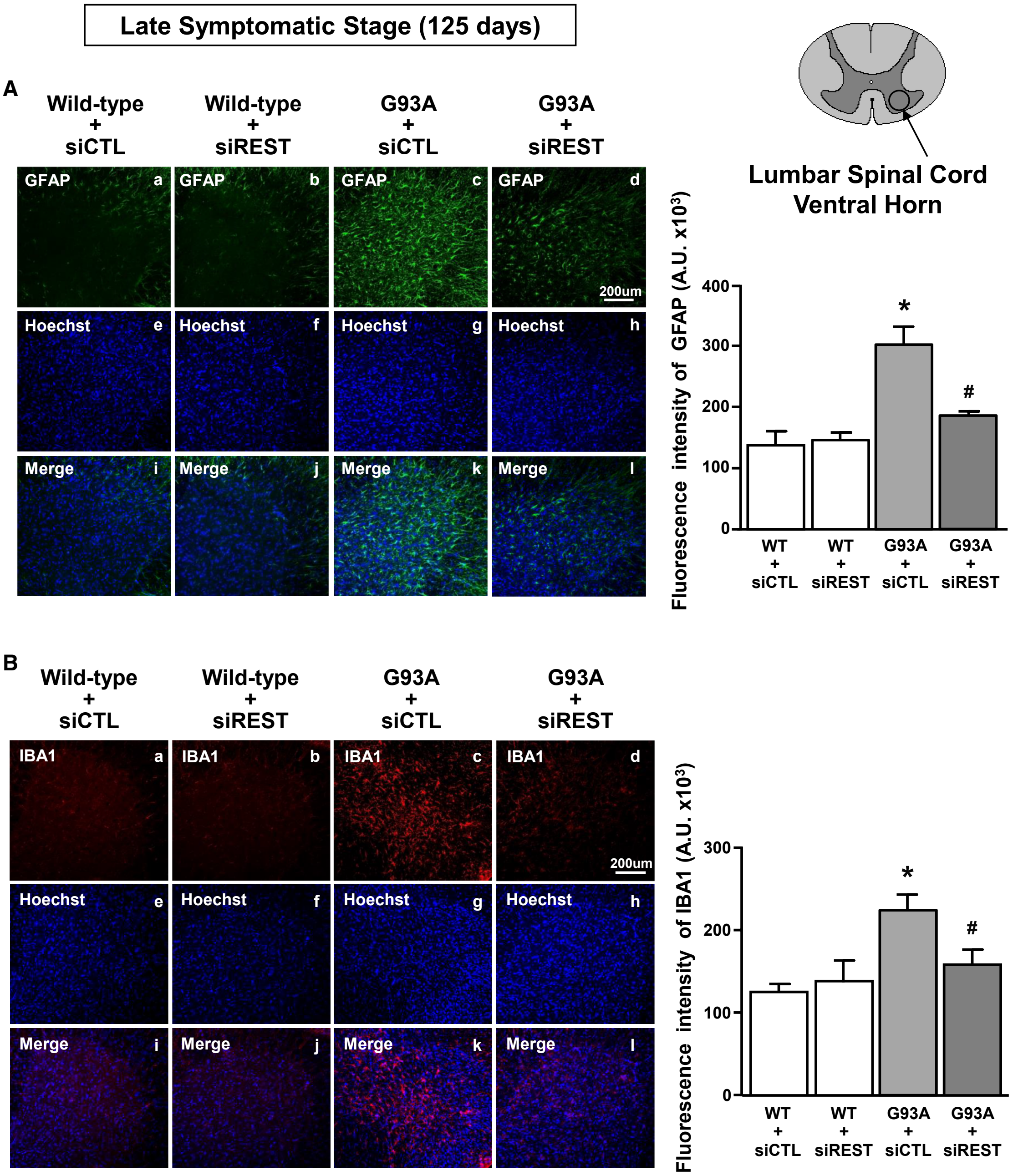

Figure 3. Effect of siREST treatment on astrocytes and microglia activation in spinal cords of fully symptomatic G93A mice

(A) Immunofluorescence analysis with quantification of fluorescence intensity of GFAP (A–D) in spinal cord sections from WT and G93A mice treated with siCTL and siREST. Nuclei were labeled with Hoechst dye (E–H). Merge images (I–L). Scale bar, 200 μm. Fluorescence intensity of GFAP expressed in A.U. (n = 3). (B) Immunofluorescence analysis with quantification of fluorescence intensity of Iba1 (A–D) in spinal cord sections from WT and G93A mice treated with siCTL and siREST. Nuclei were labeled with Hoechst dye (E–H). Merge images (I–L). Scale bar, 200 μm. Fluorescence intensity of Iba1 expressed in A.U. (n = 3). *$p < 0.05$ vs. WT + siCTL; $^{\#}p < 0.05$ vs. G93A + siCTL by one-way ANOVA analysis, followed by Bonferroni post hoc test.

Body weight

The SOD1-G93A transgenic mouse tends to show a reduction in body weight starting around the 90th day of life.[21] In siCTL-treated SOD1-G93A mice we observed a slight reduction in body weight starting in the 6th week of measurement. In ALS mice, although siREST treatment showed a trend in preventing body weight loss, especially at the 9th and 10th weeks of testing, it was not significantly effective (Figure S3).

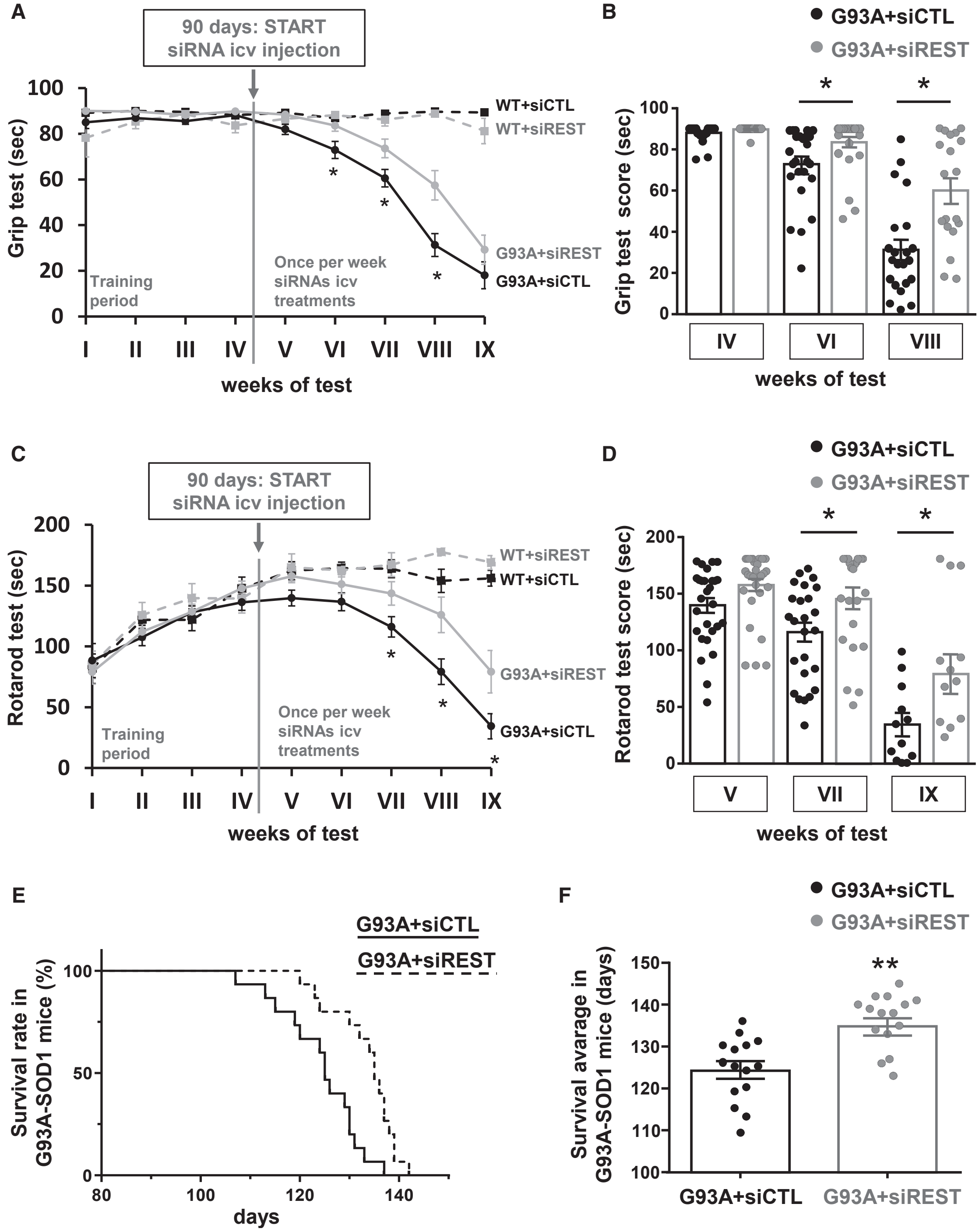

Figure 4. Effect of siREST treatment on motor behavioral performances and survival of G93A mice

Grip (A and B) and rotarod tests (C and D) of WT + siCTL (black squares, dashed line), WT + siREST (gray squares, dashed line), G93A + siCTL (gray dots, continuous line) and G93A + siREST (black dots, continuous line) mice were started at age 2 months, once per week, considering the first 2 weeks as a training period. Scattered graph plots for grip (B) and rotarod (D) tests for G93A + siCTL (black dots) and G93A + siREST (gray dots) mice every 2 weeks, starting at week 4 for grip and at week 5 for rotarod. *$p < 0.05$ vs. G93A + siCTL by two-way ANOVA analysis followed by Bonferroni test. Kaplan-Meier survival analysis, expressed as a percentage (E) or in days (F) of G93A + siCTL (continuous line and black dots; $n = 15$) and G93A + siREST (dashed line and gray dots, $n = 15$). ***$p < 0.001$ by log rank statistical test for (E) and **$p < 0.005$ by Student's t test for (F).

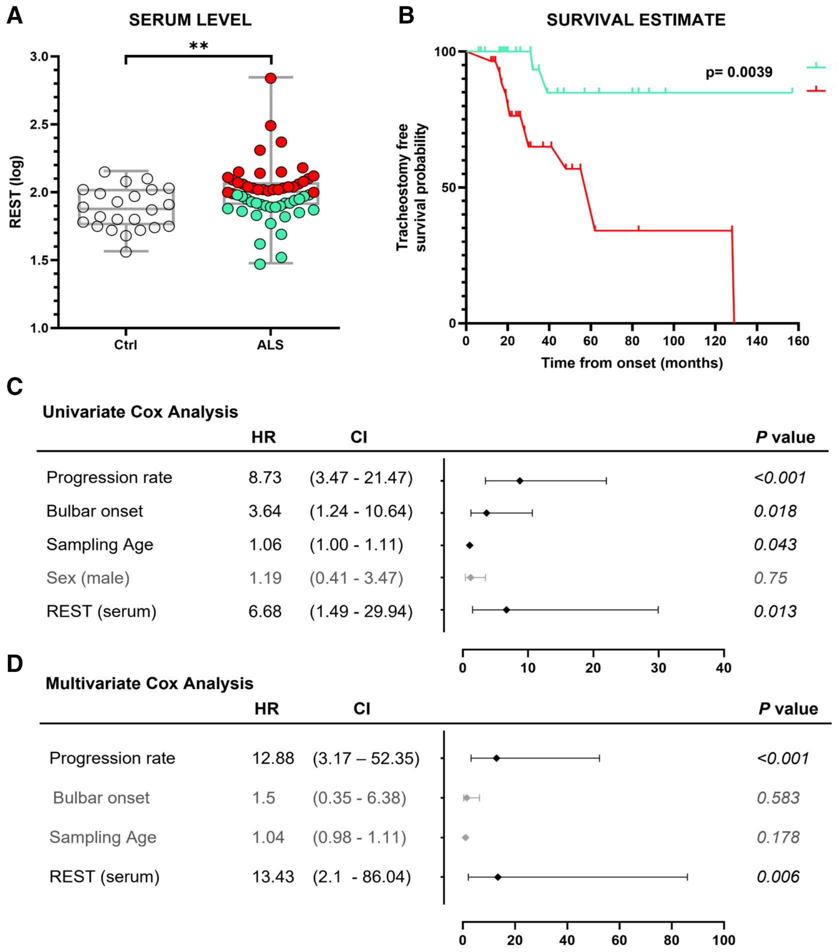

Figure 5. Serum levels and prognostic value of REST protein in ALS patients

(A) ELISA assay results showing REST protein levels in the serum of ALS patients (n = 56), compared with healthy controls (Ctrl) (n = 23). ** p < 0.01 vs. control by Student's t test. (B) Kaplan-Meier analysis of tracheostomy-free survival in ALS patients stratified according to the median serum REST protein levels. Patients with REST levels above the median, represented in red, exhibit a significantly worse prognosis than patients with levels below the median, shown in light green. (C and D) Univariate and multivariate Cox proportional hazard models assessing the prognostic value of increased serum REST levels, adjusted for bulbar onset type, sex, progression rate, and age at serum sampling. Whiskers represent the 95% confidence interval (CI). * p < 0.05, ** p < 0.01.

REST protein levels increased in the nuclear compartment of cells from the spinal cord of late symptomatic SOD1-G93A mice and its levels affected the mRNA expression of ALS-related REST target genes

Since REST can localize either in the nucleus or in the cytoplasm of cells,[22] we performed western blotting for REST in fractionated nuclear and cytosolic lysates from spinal cords of WT and G93A mice at age 125 days in order to clarify the subcellular localization of REST. Figure S4A shows that, in WT mice, REST had a strong cytosolic localization, whereas, on the contrary in late symptomatic G93A mice, REST localization was predominantly nuclear.

Moreover, we found that two ALS-related REST target genes, sodium/calcium exchanger 1 (NCX1)[23] and neuroglobin (Ngb),[24] are significantly reduced in spinal cords tissue of G93A mice at age 125 days, where a significant REST increase was observed. Coherently, siREST significantly reduced the G93A-dependent REST mRNA increase and, more importantly, counteracted the G93A-dependent NCX1 and Ngb downregulation (Figures S4B–S4D) compared with siCTL-treated mice.

Circulating plasma REST levels were elevated in ALS patients and predicted disease prognosis and survival

The final part of the present study explored the potential role of REST as a blood-based biomarker in ALS patients. To this end, we first investigated whether REST is detectable in serum and whether its levels differed between ALS patients and healthy controls (Ctrl). Using ELISA assay, we found that serum REST levels were significantly elevated in ALS patients compared with Ctrl (p = 0.006; Figure 5A).

Particularly, we examined REST concentrations across different subtypes and clinical stages. Among the ALS cohort, 4 patients (7%) carried known genetic variants (2 SOD1-G93A, 2 TARDBP), while the remaining 53 (93%) were classified as sporadic. Median serum REST levels were comparable between genetic and sporadic cases (89.5 pg/mL [interquartile range, IQR: 50.8] vs. 98.0 pg/mL [IQR: 39.8], respectively), with no statistically significant difference (Kruskal-Wallis test: H = 0.738, df = 1, p = 0.390). Similarly, REST levels did not significantly differ across King's clinical stages (H = 4.342, df = 3, p = 0.227). Median values by stage were: 109.97 pg/mL (IQR: 69.4) for stage 1, 99.87 pg/mL (IQR: 44.6) for stage 2, 83.33 pg/mL (IQR: 46.0) for stage 3, and 99.44 pg/mL (IQR: 25.2) for stage 4.

We then evaluated the prognostic risk associated with the increase of serum REST levels using univariate and multivariate Cox models. In the univariate analysis (Figure 5C), the hazard ratio (HR) for the increase in serum REST was significant (HR = 6.68; 95% confidence level [CI], 1.49–29.94; p = 0.013), while, in the multivariate model, REST remained an independent prognostic factor (HR = 13.43; 95% CI, 2.1–86.04; p = 0.006), even after adjusting for other variables such as progression rate, bulbar onset, and sampling age, highlighting its strong association with survival outcomes in ALS

(Figure 5D). Importantly, the Kaplan-Meier survival curve (Figure 5B) further supported the prognostic value of serum REST, as patients with higher REST levels exhibited significantly shorter tracheostomy-free survival compared with those with lower levels ($p = 0.0039$).

DISCUSSION

The results of our study demonstrated that the transcriptional factor REST plays a relevant role in the pathophysiology of ALS. We found a significant increase of REST in the ALS model SOD1-G93A mouse starting from the early to the final stage of the disease, in motor cortex, brainstem, and spinal cord, the brain areas affected by this neuropathology. More importantly, we demonstrated for the first time that the prevention of REST upregulation by siRNA i.c.v. injections: (1) limited the loss of the MNs, (2) reduced the formation of misfolded SOD1 aggregates, (3) mitigated the activation of astrocytic and microglial cells, (4) significantly improved muscle strength and motor coordination, and (5) prolonged the lifespan of SOD1-G93A ALS mice. Furthermore, we demonstrated that higher levels of circulating plasma REST correlated with disease severity and predicts worse prognosis in ALS patients. To sustain these results, it should be underlined that studies using bioinformatic analysis showed that genes involved in ALS, such as ALS2, ERBB4, FUS, SETX, and VAPB are particularly enriched with human-specific REST binding.[25] In addition, it should be considered that REST: (1) causes the downregulation of the neuroprotective Ngb gene in SOD1-G93A mice[24] and (2) participates in the activation of the MeHg-induced necroptotic cell death in SOD1-G93A motor neuronal-like cells.[4]

Another interesting result of the present study was that, in WT mice, REST displayed a predominant cytosolic localization, whereas in ALS mice REST was strongly present in nuclei (Figure S4A), suggesting its involvement in the transcriptional repression of its target genes. Indeed, we found a significant REST increase in the spinal cords of 125-day-old SOD1-G93A mice, whereas NCX1 and Ngb mRNA, two REST target genes that have been previously found to have a neuroprotective role in ALS,[23,24,26] were reduced. Indeed, it has been demonstrated in *in vitro* and *in vivo* ALS models that: (1) Ngb is fundamental in reactive oxygen species detoxification and it is necessary for the preservation of a normal mitochondrial phenotype, counteracting apoptotic motor neuronal death[24]; whereas (2) NCX1 by recharging endoplasmic reticulum (ER) of Ca^{2+} protects the cells from ER stress.[26] Furthermore, NCX1 pharmacological activation protects MNs from the toxic effect of β-methylamino-l-alanine (L-BMAA)[26] and prolongs life span of SOD1-G93A mice through the attenuation of MN loss.[23] In the present study we demonstrated that the knockdown of REST significantly counteracted the decrease of Ngb and NCX1 mRNA occurring in the spinal cord of symptomatic ALS mice (Figures S4C and S4D). Considering that REST has more than 2,000 putative target genes,[27] we could not exclude that other REST target genes are involved in the pathophysiology of ALS.

Similarly, it has been demonstrated that the aberrant accumulation of nuclear REST is associated with neurodegeneration in other neurologic disorders, such as stroke, Huntington's disease, and epilepsy.[27] In particular, REST increases in nuclei of: (1) hippocampal neurons in response to ischemic insults, where it causes the repression of neuroprotective genes, such as GluR2[12,28]; (2) striatal neurons, determining a reduction of BDNF in Huntington's disease[11,29]; (3) hippocampal neurons, provoking a repression of REST target genes, such as Hcn1, Kcnc2, and Calb1 in *ex vivo* and *in vivo* models of epilepsy.[30]

At variance with all the above-mentioned studies and our results, it should be mentioned that Lu et al.[31] found that in neurons within the prefrontal cortex of healthy aged individuals REST increases in the nucleus compartment, where it represses genes involved in apoptosis and oxidative stress, whereas in the brains of Alzheimer's disease (AD) patients the nuclear REST was reduced, thus contributing to neuronal death.[31] This dual role of REST could be explained by the different pathophysiological mechanisms intervening in AD and healthy aging compared with the above-mentioned neurodegenerative diseases.

Furthermore, we demonstrated that the knockdown of REST was effective in limiting a series of neurodegenerative phenomena underlying ALS, such as the loss of MNs (Figure 2A) and the formation of aggregates containing misfolded SOD1 (Figure 2B). More importantly, our behavioral and survival studies in siREST-treated ALS mice sustained the protective effect of preventing REST accumulation in ALS motor performance and survival rate. The improvement in motor skills and survival of SOD1-G93A mice treated with siREST (Figure 4) underlined that the maintenance of normal levels of REST could be fundamental for preventing neurodegenerative processes triggering ALS symptoms.

Another aspect that emerged from the present paper was that the schedule for i.c.v. siREST treatment required that the silencing administration occurred in the very early phase of the disease and continued until the late phase to be effective in improving biochemical and behavioral symptoms. Although the effect of siREST treatment in ALS mice is promising on motor performance and survival, to improve the translatability of these results further studies should be performed in the future to investigate whether these benefits could persist when siREST treatment would be administered at later stages.

In this study we also demonstrated that siREST not only exerted neuroprotection in MNs, but also on neuroinflammation, as demonstrated by the reduction of astrogliosis and microgliosis in siREST-treated G93A mice. These results are supported by the studies of other authors demonstrating that glial cells, i.e., astrocytes, oligodendrocytes, and Schwann cells,[32–36] are indirectly or directly involved in ALS. Furthermore, reactive microglia are present in ALS-affected tissues,[37] proving that the neuroinflammation may play a significant role in ALS pathophysiology.[38]

This paper ends with an interesting finding, which gives translationality to the study. It is noteworthy that the total peripheral REST plasma levels, including REST present in the neuronally derived exosomes

and the REST free-floating form, can be quantified.[13] Herein, we found that serum REST protein levels were significantly elevated in ALS patients compared with healthy controls. More intriguingly, our analysis revealed that higher serum REST levels were associated with a worse prognosis in ALS patients, as reflected by the reduced tracheostomy-free survival in individuals with elevated REST levels.

This relationship between ALS progression and REST serum increase does not occur in other neurodegenerative disease, such as AD; in fact, total circulating REST plasma levels declined with increasing severity of AD risk and impairment,[13,14] suggesting a distinct role of the putative biomarker REST in ALS compared with AD patients. A limitation of the present study is that plasma REST levels were only compared between ALS patients and healthy controls. Therefore, we cannot determine whether the increase in REST is specific to ALS or reflects a broader marker of neurodegeneration. This divergence highlights the potential disease-specific behavior of REST and supports the need for future comparative studies to explore its utility as a differential biomarker across neurodegenerative disorders.

Another aspect that deserves to be discussed is that the secretion mechanism of REST is not well characterized. However, previous reports have demonstrated by ELISA assay that other nuclear proteins, such as TAR DNA-binding protein 43 (TDP-43), are augmented in blood of ALS patients,[39] as well as transcription factor HIF-1 in plasma and sera of ischemic patients.[40,41] Furthermore, at least two different studies demonstrated that REST is detectable in the blood and that the reduced circulating REST levels in the plasma of AD participants mirrors the brain REST reduction in autopsy-confirmed AD patients.[13,14] Moreover, we acknowledge that REST is classically characterized as a nuclear transcriptional repressor, and that its extracellular presence may arise through non-canonical mechanisms activated during neurodegenerative processes, such as passive release during cell death and vesicle-mediated export. It is plausible that REST may be released under similar circumstances in ALS and that the increased circulating REST in ALS participants could mirror the augmented REST expression in CNS of ALS patients, as we observed in the CNS of ALS mice. Notably, plasma samples were pretreated with Tween 20 (4% v/v) to stimulate the release of REST contained within extracellular vesicles.[13] Thus, the total peripheral REST plasma levels that we measured include the amount derived from exosomes and the REST free-floating form. Moreover, it is necessary to clarify that REST can be expressed in multiple cell types, such as glial or immune cells,[42,43] and we cannot exclude that a portion of REST we measured in the serum derives from non-neuronal cells.

Conclusions

Collectively, the results of this study provide novel insights, showing for the first time that:

(1) REST plays a pivotal role in ALS pathophysiology;
(2) Preventing REST accumulation reduces motor neuronal death and ameliorates symptoms in transgenic ALS models, and may help to develop new therapeutic strategies;
(3) Circulating serum REST levels correlate with disease progression in ALS patients, reinforcing its potential as a clinically relevant biomarker.

Given the lack of effective therapies and reliable biomarkers for ALS, our findings suggest that pharmacological inhibition of REST and routine assessment of circulating serum REST levels might represent innovative strategies for both treatment and early diagnosis/prognosis of ALS. Further research is warranted to explore the therapeutic potential of targeting REST and to validate its utility as biomarker in larger, independent ALS cohorts.

METHODS

Animal care and use

Experiments were performed on male transgenic mice B6SJL-TgN (SOD1G93A)1Gur over-expressing human SOD1, containing the Gly93 to Ala (G93A) mutation (Jackson Laboratory, stock no. 002726); these mice have high transgene copy number, as reported in the datasheet. The colony was derived by breeding male transgenic (TG) mice to naive (B6xSJL/J)F1 females (WT) (Janvier SAS). Overall, 40 WT and 35 TG mice, housed under diurnal lighting conditions (12 h darkness/light) were used. All experimental procedures on live animals were performed according to the international guidelines for animal research and approved by the Animal Care Committee of "Federico II" University of Naples, Italy and Ministry of Health, Italy. All efforts were made to minimize the number of animals used and their suffering. They were identified by PCR according to Jackson Laboratory's genotyping protocol.

Genotyping mice

DNA from mouse tails was extracted as described previously.[23] On the extracted DNA, we performed PCR in order to evaluate the presence of the human transgene superoxide dismutase-1 (hSOD1) gene, and these mice were referred as G93A. The primers used were: hSOD1 fwd 5′-CATCAGCCCTAATCCATCTGA-3′ and hSOD1 rev 5′-CGCGACTAACAATCAAAGTGA-3′.

RT-PCR analysis

Mice were deeply anesthetized with 3% isoflurane vaporized in O_2/N_2O 50:50 and sacrificed. Tissues were quickly removed, then immediately frozen on dry ice and stored at −80°C until use. Total RNA was extracted with TRIzol, following supplier's instructions (Thermo Fisher, Milan, Italy) and cDNA was synthesized using 2 μg of total RNA with the High Capacity Transcription Kit following the supplier's instruction (Thermo Fisher). Quantitative real-time PCR with TaqMan assays for NCX1 (ID: Mm00441524_m1), REST (ID: Mm00803268_m1), Ngb (ID: (Mm00452101_m1) genes, and glucuronidase β (Gusb, ID: Mm00446953_m1) as housekeeping were performed in a 7500 real-time PCR system (Applied Biosystems). Samples were amplified simultaneously in triplicate in one assay run. Changes in mRNA levels were determined as the difference in threshold cycle ($2^{-\Delta\Delta CT}$) between the target gene and the reference gene.

Western blotting and Co-immunoprecipitation assay

Experiments for Western blotting, Co-immunoprecipitation and nucleus/cytosol fractionation were performed as reported previously.[10] Proteins for all experiments were separated on 4%–15% precast polyacrylamide gel (Bio-Rad) and then transferred onto nitrocellulose membranes using the Trans-Blot SD Semi-Dry Transfer Cell (Bio-Rad). Membranes were blocked with EveryBlot Blocking Buffer (Bio-Rad, catalog no. 12010020) for 2 h at room temperature, and then incubated overnight at 4°C with 1:1,000 anti-REST (Merck Millipore, catalog no. 07-579) and then 1:5,000 anti-α-tubulin (Sigma-Aldrich, catalog no. T5168), which has been used for normalization.

For nucleus/cytosol fractionation spinal cords from age 125 days mice were mechanically shredded. The cell pellet was resuspended in 500 μL of hypotonic buffer (10 mM HEPES KOH [pH 7.9], 10 mM KCl, 1.5 mM $MgCl_2$, 0.1 mM EGTA, 0.5 mM DTT, 1 mM NaV_3O_4, and 0.2 mM PMSF) and incubated on ice for 15 min. Then cells were centrifuged at 3,000 rpm for 5 min and the pellet was resuspended in hypotonic buffer. Next, cells were passed 8–10 times through a 1-mL syringe. The homogenate was centrifuged for 10 min at 1,000 rpm at 4°C to obtain the cytoplasmic fraction (supernatant) and nuclear fraction (pellet). The nuclear pellet was resuspended in 50 μL of complete cell extraction buffer (20 mM HEPES KOH [pH 7.9], 0.4 M NaCl, 1.5 mM $MgCl_2$, 0.1 mM EGTA, 25% glycerol, 0.5 mM DTT, 1 mM NaV_3O_4, 0.2 mM PMSF, 0.5% deoxycholate) and incubated on ice for 30 min with vortexing at 10-min intervals. The nuclear lysate was centrifuged at 14,000 rpm for 30 min at 4°C to obtain the nuclear fraction (supernatant). Anti-histone H3 was used to assess the nuclear extracts purity; anti-β-actin (Sigma-Aldrich, catalog no. A3854) was used as loading control.

For IP assay, 1,500 μg of protein lysates from spinal cords of age 125 days mice were immunoprecipitated overnight at 4°C using 3 μg of misfolded SOD1 mouse monoclonal antibody B8H10 (MediMab, catalog no. MM-0070-P) or normal IgG, as negative control. Fifty micrograms of pre-immunoprecipitated lysates were used as input; anti-α-tubulin was used as loading control. The immunoprecipitates were then subjected to western blot analysis as described above. Membranes were incubated overnight at 4°C with 1:1,000 anti-SOD1 (Invitrogen, catalog no. PA1-30195).

Stereotaxic surgery and administration of siRNAs

Male and female G93A or WT mice were positioned on a stereotaxic frame, and a 26-g stainless steel guide cannula was implanted into the right lateral ventricle using the following stereotaxic coordinates from the bregma: 0.3 mm caudal, 1 mm lateral, and 2 mm below the dura. siREST or siCTL (5 μL, 10 μM) were i.c.v. administered starting from day 90 of life, once a week, for 4 consecutive weeks.

Tissue processing, immunostaining, and confocal immunofluorescence

Animals were anesthetized and transcardially perfused with saline solution containing 0.01 mL heparin, followed by 4% paraformaldehyde in 0.1 mol/L PBS saline solution. Brains and spinal cords were processed as described previously.[23] Spinal cords were rapidly removed on ice and postfixed overnight at +4°C and cryoprotected in 30% sucrose in 0.1 M phosphate buffer (PB) with sodium azide 0.02% for 24 h at 4°C. Next, spinal cords and brains were sectioned frozen on a sliding cryostat at 40 μm thickness in the rostrum-caudal direction. Afterward, free-floating serial sections were incubated with PB Triton X-0.3% and blocking solution (0.5% milk, 10% FBS, 1% BSA) for 1 h and 30 min. The sections were incubated overnight at +4°C with the following primary antibodies: anti-SMI32 (BioLegend, catalog no. SMI-32P), anti-REST (Merck Millipore, catalog no. 07-759), anti-GFAP (Abcam, catalog no. AB7260), and anti-IBA1 (Wako, catalog no. 019-19,741). The sections were then incubated with the corresponding fluorescent-labeled secondary antibodies, Alexa 488/Alexa 594-conjugated antimouse/antirabbit IgG. Nuclei were counter-stained with Hoechst. Images were observed using a Zeiss LSM700 META/laser scanning confocal microscope (Zeiss, Oberkochen, Germany). Single images were taken at a resolution of 1,024 × 1,024. Nissl staining was performed as described previously.[23] Briefly, slide-mounted sections were dipped for 7 min in 0.5% solution of cresyl violet in distilled water supplemented with acetic acid (16 N solution, 60 drops/L). Slides were then rinsed in distilled water, dehydrated through graded ethanol baths (95%, 100%; 5 min each), dilapidated for 8 min in xylene, and coverslipped with Eukitt Mounting Medium.

GFAP/IBA1 analysis

Frozen spinal cord was sectioned on a sliding cryostat at 20 μm. In lumbar spinal cord, images from the same areas of each spinal cord region were compared. Analyses were performed using ImageJ software in the total number of positive signals of GFAP antibody for photographic field (mm^2) in lumbar spinal cord (L1-L6) of mice. $n = 3$ mice per treatment group and 5/6 sections were analyzed.

MN counting analysis

MNs were counted in the ventral horns of the lumbar spinal cord. Sections of each area were analyzed as described previously.[23] Frozen brain tissue and spinal cord were sectioned on a sliding cryostat at 20 μm in the rostrum-caudal direction. Analyses were performed using ImageJ software in polygonal-shaped neurons larger than 150–200 μm^2 with a well-defined cytoplasm, nucleus, and nucleolus for MN counting. Quantification of MNs was determined by counting and averaging 3/4 sections selected at equally spaced intervals spanning L1-L6 under 20× magnification, $n = 3$ mice for each experimental group, and 5/6 sections were analyzed. Cell counting analysis was determined as the total MNs per field (mm^2) of mice.

Behavioral assessment

In order to follow the disease progression, G93A mice underwent specific behavioral tests: rotarod and paw grip endurance (Grip) tests were performed by a trained blind observer, as reported previously,[44] starting from postnatal day 60 (P60), a fully asymptomatic phase of the disease. The first 4 weeks of tests were considered as training for the animals. The tests were performed once/week. The body weight was also monitored during the whole period of observation. Briefly, for

the rotarod test we measured the time that animals could remain on the rotating cylinder in a 7650 accelerating model of a rotarod apparatus (Ugo Basile, Italy). Each animal was given three trials. The arbitrary cut-off time was 180 s, and the accelerated speed went from 4 to 15 rpm. For Grip test the animal was placed on the wire-lid of conventional housing cage: the lid was gently shaken to prompt the mouse to hold onto the grid before it was swiftly turned upside-down. Grip score was measured as the length of time that the mouse was able to hang on to the grid. The arbitrary cut-off time was 90 s.

Human participants and clinical characterization

This prospective longitudinal study included 79 participants who were evaluated at the Department of Neurology and the ALS Center of the University Federico II of Naples, Italy, between January 2022 and May 2024. The study cohort comprised 56 male and female patients diagnosed with ALS (52 sporadic ALS; 4 familial ALS) and 23 male and female healthy controls, recruited from the caregivers of patients.

Patients with ALS were diagnosed according to the revised El Escorial criteria, meeting the classification of "possible," "probable," "probable laboratory-supported," or "definite" ALS.[45] In addition, each patient underwent a complete neurological and neurophysiological examination for diagnosis. Disease severity was assessed using the revised ALS Functional Rating Scale (ALSFRS-R)[46] and respiratory function was assessed through spirometry with the patient sitting upright.[47] We then computed the ALSFRS-R progression rate[48] using the following formula: (48 − ALSFRS-R score at baseline assessment)/(months from onset to assessment). Exclusion criteria were: (1) significant neurological or psychiatric illness other than ALS and (2) significant unstable systemic illness or organ failure.

All ALS patients underwent genetic screening for the four most common ALS-related genes: C9orf72 repeat expansion, and mutations in SOD1, TARDBP, and FUS. Clinical evaluations and REST analysis were performed across the entire ALS cohort, encompassing both sporadic and genetic cases. Written informed consent was obtained from all participants in accordance with the Declaration of Helsinki before study enrollment. The study protocol was approved by the local Ethics Committee (protocol nos. 100/17/ES01 and 151/2023).

Serum REST protein and immunological assay

Before obtaining a blood sample, participants were required to fast for 8 h. After collection, blood samples were centrifuged at $3,000 \times g$ for 10 min at 4°C within 2 h of collection. Serum fraction was separated and stored in aliquots and then kept frozen at −80°C until further use. Serum samples were kept at room temperature for 30 min, then were pretreated with Tween 20 (4% v/v) to release proteins contained within extracellular vesicles (i.e., exosomes) and mixed on an orbital shaker for 15 min, finally they were processed according to the product instructions. Serum REST level was quantified by a human-specific ELISA kit specific for REST (Cusabio, American Research Products, Waltham, MA). This assay employs the quantitative sandwich enzyme immunoassay technique. Antibody specific for REST is pre-coated onto a microplate. Standards and samples are pipetted into the wells and any REST present is bound by the immobilized antibody. After removing any unbound substances, biotin-conjugated horseradish peroxidase is added to the wells. Following a wash to remove any unbound avidin-enzyme reagent, a substrate solution is added to the wells and color develops in proportion to the amount of REST bound in the initial step. The color development is stopped before saturation and the intensity of the color measured. Serum samples were measured in duplicate and the mean absorbance value (450 nm) was considered for the analysis. The investigator who performed the ELISA assay was blinded to the group allocation. For the calculation of results, the data were linearized by plotting the log of the REST concentrations vs. the log of the optical density (OD), as suggested by the producers.

Technical specifications of the ELISA assay kit

Detection range of the assay

The detection range of the assay was 31.25–2,000 pg/mL.

Sensitivity

The minimum detectable dose of human REST is typically less than 7.81 pg/mL. The sensitivity of this assay, or lower limit of detection was defined as the lowest protein concentration that could be differentiated from zero. It was determined that the mean OD value of 20 replicates of the zero standard added by their three standard deviations.

Specificity

This assay has high specificity for detection of human REST. No significant cross-reactivity or interference between human REST and analogs was observed.

Precision

Intra-assay precision: CV% <8%. Three samples of known concentration were tested 20 times on one plate for assessment. Inter-assay precision: CV% <10%. Three samples of known concentration were tested in 20 different assays to assess.

Serum samples were measured in duplicate and the mean absorbance value (450 nm) was considered for the analysis. The investigator who performed the ELISA assay was blinded to the group allocation.

Statistical analysis

Animal samples

Data were analyzed using GraphPad Prism 5 software (Graph Pad Software). All bars in the figures represent the mean ± SD. Statistical differences between two experimental groups were analyzed using Student's t test. Statistically significant differences for single time point comparisons between multiple groups were evaluated by one-way ANOVA, followed by Bonferroni's post hoc test, whereas a two-way ANOVA, followed by Bonferroni post hoc test, was used to analyze the significance between multiple groups in different time points. Statistical significance was accepted at the 95% CI ($p < 0.05$).

Human samples

Demographic and clinical characteristics of human participants are reported as percentages for categorical variables and as medians with IQRs for non-parametric continuous variables. Demographic and REST levels were compared between the two groups (ALS vs. Ctrl) using the χ^2 test for categorical variables and the Mann-Whitney test for continuous variables.

Univariate and multivariate analyses for tracheostomy-free survival (time from disease onset to tracheostomy or death) were conducted using the Cox proportional hazards model, with p values calculated via the Wald test. The Cox model included the following covariates: age, sex, site of disease onset (spinal vs. bulbar), disease progression rate (ALSFRS-R_rate), and REST levels stratified by the median value.

Tracheostomy-free survival curves were constructed using the Kaplan-Meier method, and the log rank test was employed to assess differences between groups based on the median REST levels. Statistical analyses were performed using SPSS version 29.0 (SPSS, Chicago, IL). Bonferroni correction was applied to adjust for alpha inflation due to multiple comparisons where appropriate.

DATA AND CODE AVAILABILITY

Data are available upon request to the corresponding authors.

ACKNOWLEDGMENTS

This work was supported by the following grants: PNRR, Spoke 3, MNESYS, PE00000006 M4 C2 I 1.3, finanziato dall'Unione Europea- NextGeneration EU- CUP: E63C22002170007 (to L.F., N.G. and G.P.), PNRR, Spoke 7, MNESYS, PE00000006, M4 C2 I 1.3, finanziato dall' Unione Europea- NextGenerationEU- CUP: B63D22000600006 (to L.A. and N.D.I.), PRIN 2022, project 0225BMTWJ to V.V.; PRIN 2022 PNRR, project P2022WPRKA to V.V.; RAREGLIALS, CUP: E63C23002410002 ("Sviluppo di strategie terapeutiche e diagnostiche innovative per la SMA, la SLA e il Glioblastoma mediante l'identificazione di meccanismi Genetici, epigenetici e molecolari condivisi" to L.A.) and from the Italian Ministry of Health (Ricerca Corrente Project) (to L.A., and N.D.I.).

AUTHOR CONTRIBUTIONS

Conception and design, N.G., L.F., and L.A.; development of methodology, N.G., V.V., S.A., R.D., O.C., S.R., G.S., V.V.I., X.K., N.D.I., and G.P.; acquisition of data, N.G., V.V., S.A., R.D., O.C., S.R., G.S., V.V.I., X.K., N.D.I., and G.P.; analysis and interpretation of data, N.G., V.V., S.A., R.D., O.C., and S.R.; writing – original draft, N.G., L.F., R.D., and L.A.; supervision of study, N.G., L.F., R.D., and L.A.

DECLARATION OF INTERESTS

The authors declare no competing interests.

SUPPLEMENTAL INFORMATION

Supplemental information can be found online at https://doi.org/10.1016/j.ymthe.2025.10.039.

REFERENCES

1. Geevasinga, N., Menon, P., Ozdinler, P.H., Kiernan, M.C., and Vucic, S. (2016). Pathophysiological and diagnostic implications of cortical dysfunction in ALS. Nat. Rev. Neurol. *12*, 651. https://doi.org/10.1038/nrneurol.2016.140.
2. Tokuda, E., Okawa, E., and Ono, S.I. (2009). Dysregulation of intracellular copper trafficking pathway in a mouse model of mutant copper/zinc superoxide dismutase-linked familial amyotrophic lateral sclerosis. J. Neurochem. *111*, 181–191. https://doi.org/10.1111/j.1471-4159.2009.06310.x.
3. Orrell, R.W. (2000). Amyotrophic lateral sclerosis: copper/zinc superoxide dismutase (SOD1) gene mutations. Neuromuscul. Disord. *10*, 63–68. https://doi.org/10.1016/s0960-8966(99)00071-1.
4. Guida, N., Sanguigno, L., Mascolo, L., Calabrese, L., Serani, A., Molinaro, P., Lau, C.G., Annunziato, L., and Formisano, L. (2021). The Transcriptional Complex Sp1/KMT2A by Up-Regulating Restrictive Element 1 Silencing Transcription Factor Accelerates Methylmercury-Induced Cell Death in Motor Neuron-Like NSC34 Cells Overexpressing SOD1-G93A. Front. Neurosci. *15*, 771580. https://doi.org/10.3389/fnins.2021.771580.
5. Su, X.J., Shen, B.D., Wang, K., Song, Q.X., Yang, X., Wu, D.S., Shen, H.X., and Zhu, C. (2022). Roles of the Neuron-Restrictive Silencer Factor in the Pathophysiological Process of the Central Nervous System. Front. Cell Dev. Biol. *10*, 834620. https://doi.org/10.3389/fcell.2022.834620.
6. Prestigio, C., Ferrante, D., Marte, A., Romei, A., Lignani, G., Onofri, F., Valente, P., Benfenati, F., and Baldelli, P. (2021). REST/NRSF drives homeostatic plasticity of inhibitory synapses in a target-dependent fashion. Elife *10*, e69058. https://doi.org/10.7554/eLife.69058.
7. Baldelli, P., and Meldolesi, J. (2015). The Transcription Repressor REST in Adult Neurons: Physiology, Pathology, and Diseases. eNeuro *2*, ENEURO.0010-15.2015. https://doi.org/10.1523/ENEURO.0010-15.2015.
8. McClelland, S., Brennan, G.P., Dubé, C., Rajpara, S., Iyer, S., Richichi, C., Bernard, C., and Baram, T.Z. (2014). The transcription factor NRSF contributes to epileptogenesis by selective repression of a subset of target genes. Elife *3*, e01267.
9. Morris-Blanco, K.C., Kim, T., Bertogliat, M.J., Mehta, S.L., Chokkalla, A.K., and Vemuganti, R. (2019). Inhibition of the Epigenetic Regulator REST Ameliorates Ischemic Brain Injury. Mol. Neurobiol. *56*, 2542–2550. https://doi.org/10.1007/s12035-018-1254-y.
10. Guida, N., Serani, A., Sanguigno, L., Mascolo, L., Cuomo, O., Fioriniello, S., Marano, D., Ragione, F.D., Anzilotti, S., Brancaccio, P., et al. (2024). Stroke Causes DNA Methylation at Ncx1 Heart Promoter in the Brain Via DNMT1/MeCP2/REST Epigenetic Complex. J. Am. Heart Assoc. *13*, e030460. https://doi.org/10.1161/jaha.123.030460.
11. Zuccato, C., Tartari, M., Crotti, A., Goffredo, D., Valenza, M., Conti, L., Cataudella, T., Leavitt, B.R., Hayden, M.R., Timmusk, T., et al. (2003). Huntingtin interacts with REST/NRSF to modulate the transcription of NRSE-controlled neuronal genes. Nat. Genet. *35*, 76–83.
12. Calderone, A., Jover, T., Noh, K.M., Tanaka, H., Yokota, H., Lin, Y., Grooms, S.Y., Regis, R., Bennett, M.V.L., and Zukin, R.S. (2003). Ischemic insults derepress the gene silencer REST in neurons destined to die. J. Neurosci. *23*, 2112–2121.
13. Ashton, N.J., Hye, A., Leckey, C.A., Jones, A.R., Gardner, A., Elliott, C., Wetherell, J.L., Lenze, E.J., Killick, R., and Marchant, N.L. (2017). Plasma REST: a novel candidate biomarker of Alzheimer's disease is modified by psychological intervention in an at-risk population. Transl. Psychiatry *7*, e1148. https://doi.org/10.1038/tp.2017.113.
14. Wei, M., Ni, J., Shi, J., Li, T., Xu, X., Li, C., Qin, B., Fan, D., Xie, H., Wang, Z., et al. (2022). Plasma repressor element 1-silencing transcription factor levels are decreased in patients with Alzheimer's disease. BMC Geriatr. *22*, 471. https://doi.org/10.1186/s12877-022-03163-8.
15. Liu, H.N., Tjostheim, S., Dasilva, K., Taylor, D., Zhao, B., Rakhit, R., Brown, M., Chakrabartty, A., McLaurin, J., and Robertson, J. (2012). Targeting of monomer/misfolded SOD1 as a therapeutic strategy for amyotrophic lateral sclerosis. J. Neurosci. *32*, 8791–8799. https://doi.org/10.1523/jneurosci.5053-11.2012.
16. Tokuda, E., Takei, Y.I., Ohara, S., Fujiwara, N., Hozumi, I., and Furukawa, Y. (2019). Wild-type Cu/Zn-superoxide dismutase is misfolded in cerebrospinal fluid of sporadic amyotrophic lateral sclerosis. Mol. Neurodegener. *14*, 42. https://doi.org/10.1186/s13024-019-0341-5.
17. Leyton-Jaimes, M.F., Benaim, C., Abu-Hamad, S., Kahn, J., Guetta, A., Bucala, R., and Israelson, A. (2016). Endogenous macrophage migration inhibitory factor reduces the accumulation and toxicity of misfolded SOD1 in a mouse model of ALS. Proc. Natl. Acad. Sci. USA *113*, 10198–10203. https://doi.org/10.1073/pnas.1604600113.
18. Clarke, B.E., and Patani, R. (2020). The microglial component of amyotrophic lateral sclerosis. Brain *143*, 3526–3539. https://doi.org/10.1093/brain/awaa309.

19. Tripathi, P., Rodriguez-Muela, N., Klim, J.R., de Boer, A.S., Agrawal, S., Sandoe, J., Lopes, C.S., Ogliari, K.S., Williams, L.A., Shear, M., et al. (2017). Reactive Astrocytes Promote ALS-like Degeneration and Intracellular Protein Aggregation in Human Motor Neurons by Disrupting Autophagy through TGF-β1. Stem Cell Rep. *9*, 667–680. https://doi.org/10.1016/j.stemcr.2017.06.008.

20. Brooks, S.P., and Dunnett, S.B. (2009). Tests to assess motor phenotype in mice: a user's guide. Nat. Rev. Neurosci. *10*, 519–529. https://doi.org/10.1038/nrn2652.

21. Kitaoka, Y., Seki, S., Kawata, S., Nishiura, A., Kawamura, K., Hiraoka, S.I., Kogo, M., and Tanaka, S. (2023). Analysis of Feeding Behavior Characteristics in the Cu/Zn Superoxide Dismutase 1 (SOD1) SOD1G93A Mice Model for Amyotrophic Lateral Sclerosis (ALS). Nutrients *15*, 1651. https://doi.org/10.3390/nu15071651.

22. Zhao, Y., Zhu, M., Yu, Y., Qiu, L., Zhang, Y., He, L., and Zhang, J. (2017). Brain REST/NRSF Is Not Only a Silent Repressor but Also an Active Protector. Mol. Neurobiol. *54*, 541–550. https://doi.org/10.1007/s12035-015-9658-4.

23. Anzilotti, S., Valsecchi, V., Brancaccio, P., Guida, N., Laudati, G., Tedeschi, V., Petrozziello, T., Frecentese, F., Magli, E., Hassler, B., et al. (2021). Prolonged NCX activation prevents SOD1 accumulation, reduces neuroinflammation, ameliorates motor behavior and prolongs survival in a ALS mouse model. Neurobiol. Dis. *159*, 105480. https://doi.org/10.1016/j.nbd.2021.105480.

24. Van Acker, Z.P., Declerck, K., Luyckx, E., Vanden Berghe, W., and Dewilde, S. (2019). Non-Methylation-Linked Mechanism of REST-Induced Neuroglobin Expression Impacts Mitochondrial Phenotypes in a Mouse Model of Amyotrophic Lateral Sclerosis. Neuroscience *412*, 233–247. https://doi.org/10.1016/j.neuroscience.2019.05.039.

25. Rockowitz, S., and Zheng, D. (2015). Significant expansion of the REST/NRSF cistrome in human versus mouse embryonic stem cells: potential implications for neural development. Nucleic Acids Res. *43*, 5730–5743. https://doi.org/10.1093/nar/gkv514.

26. Petrozziello, T., Boscia, F., Tedeschi, V., Pannaccione, A., de Rosa, V., Corvino, A., Severino, B., Annunziato, L., and Secondo, A. (2022). Na(+)/Ca(2+) exchanger isoform 1 takes part to the Ca(2+)-related prosurvival pathway of SOD1 in primary motor neurons exposed to beta-methylamino-L-alanine. Cell Commun. Signal. *20*, 8. https://doi.org/10.1186/s12964-021-00813-z.

27. Hwang, J.Y., and Zukin, R.S. (2018). REST, a master transcriptional regulator in neurodegenerative disease. Curr. Opin. Neurobiol. *48*, 193–200. https://doi.org/10.1016/j.conb.2017.12.008.

28. Noh, K.M., Hwang, J.Y., Follenzi, A., Athanasiadou, R., Miyawaki, T., Greally, J.M., Bennett, M.V.L., and Zukin, R.S. (2012). Repressor element-1 silencing transcription factor (REST)-dependent epigenetic remodeling is critical to ischemia-induced neuronal death. Proc. Natl. Acad. Sci. USA *109*, E962–E971.

29. Zuccato, C., Belyaev, N., Conforti, P., Ooi, L., Tartari, M., Papadimou, E., MacDonald, M., Fossale, E., Zeitlin, S., Buckley, N., and Cattaneo, E. (2007). Widespread disruption of repressor element-1 silencing transcription factor/neuron-restrictive silencer factor occupancy at its target genes in Huntington's disease. J. Neurosci. *27*, 6972–6983. https://doi.org/10.1523/jneurosci.4278-06.2007.

30. Hall, A.M., Kamei, N., Shao, M., Mun, H.S., Chen, K., Chen, Y., and Baram, T.Z. (2024). Inhibition of Neuron-Restrictive Silencing Factor (REST/NRSF) Chromatin Binding Attenuates Epileptogenesis. eNeuro *11*, ENEURO.0006-24.2024. https://doi.org/10.1523/eneuro.0006-24.2024.

31. Lu, T., Aron, L., Zullo, J., Pan, Y., Kim, H., Chen, Y., Yang, T.-H., Kim, H.-M., Drake, D., Liu, X.S., et al. (2014). REST and stress resistance in ageing and Alzheimer/'s disease. Nature *507*, 448–454. https://doi.org/10.1038/nature13163. http://www.nature.com/nature/journal/v507/n7493/abs/nature13163.html#supplementary-information.

32. Philips, T., and Rothstein, J.D. (2015). Rodent Models of Amyotrophic Lateral Sclerosis. Curr. Protoc. Pharmacol. *69*, 5.67.1–5.67.21. https://doi.org/10.1002/0471141755.ph0567s69.

33. Boillée, S., Yamanaka, K., Lobsiger, C.S., Copeland, N.G., Jenkins, N.A., Kassiotis, G., Kollias, G., and Cleveland, D.W. (2006). Onset and progression in inherited ALS determined by motor neurons and microglia. Science *312*, 1389–1392. https://doi.org/10.1126/science.1123511.

34. Lobsiger, C.S., Boillee, S., McAlonis-Downes, M., Khan, A.M., Feltri, M.L., Yamanaka, K., and Cleveland, D.W. (2009). Schwann cells expressing dismutase active mutant SOD1 unexpectedly slow disease progression in ALS mice. Proc. Natl. Acad. Sci. USA *106*, 4465–4470. https://doi.org/10.1073/pnas.0813339106.

35. Nagai, M., Re, D.B., Nagata, T., Chalazonitis, A., Jessell, T.M., Wichterle, H., and Przedborski, S. (2007). Astrocytes expressing ALS-linked mutated SOD1 release factors selectively toxic to motor neurons. Nat. Neurosci. *10*, 615–622. https://doi.org/10.1038/nn1876.

36. Trotti, D., Rolfs, A., Danbolt, N.C., Brown, R.H., Jr., and Hediger, M.A. (1999). SOD1 mutants linked to amyotrophic lateral sclerosis selectively inactivate a glial glutamate transporter. Nat. Neurosci. *2*, 427–433. https://doi.org/10.1038/8091.

37. Robberecht, W., and Philips, T. (2013). The changing scene of amyotrophic lateral sclerosis. Nat. Rev. Neurosci. *14*, 248–264. https://doi.org/10.1038/nrn3430.

38. Ferraiuolo, L., Kirby, J., Grierson, A.J., Sendtner, M., and Shaw, P.J. (2011). Molecular pathways of motor neuron injury in amyotrophic lateral sclerosis. Nat. Rev. Neurol. *7*, 616–630. https://doi.org/10.1038/nrneurol.2011.152.

39. Verstraete, E., Kuiperij, H.B., van Blitterswijk, M.M., Veldink, J.H., Schelhaas, H.J., van den Berg, L.H., and Verbeek, M.M. (2012). TDP-43 plasma levels are higher in amyotrophic lateral sclerosis. Amyotroph. Lateral Scler. *13*, 446–451. https://doi.org/10.3109/17482968.2012.703208.

40. Amalia, L., Sadeli, H.A., Parwati, I., Rizal, A., and Panigoro, R. (2020). Hypoxia-inducible factor-1α in acute ischemic stroke: neuroprotection for better clinical outcome. Heliyon *6*, e04286. https://doi.org/10.1016/j.heliyon.2020.e04286.

41. Cai, Y., Zhuang, Y.K., Wu, X.Y., Dong, X.Q., Du, Q., Yu, W.H., Wang, K.Y., Hu, W., and Zheng, Y.K. (2021). Serum Hypoxia-Inducible Factor 1alpha Levels Correlate with Outcomes After Intracerebral Hemorrhage. Ther. Clin. Risk Manag. *17*, 717–726. https://doi.org/10.2147/tcrm.s313433.

42. Abrajano, J.J., Qureshi, I.A., Gokhan, S., Zheng, D., Bergman, A., and Mehler, M.F. (2009). REST and CoREST modulate neuronal subtype specification, maturation and maintenance. PLoS One *4*, e7936. https://doi.org/10.1371/journal.pone.0007936.

43. Wang, G., Yang, X., Qi, M., Li, M., Dong, M., Xu, R., and Zhang, C. (2023). Systematic analysis identifies REST as an oncogenic and immunological biomarker in glioma. Sci. Rep. *13*, 3023. https://doi.org/10.1038/s41598-023-30248-0.

44. Valsecchi, V., Boido, M., Montarolo, F., Guglielmotto, M., Perga, S., Martire, S., Cutrupi, S., Iannello, A., Gionchiglia, N., Signorino, E., et al. (2020). The transcription factor Nurr1 is upregulated in amyotrophic lateral sclerosis patients and SOD1-G93A mice. Dis. Model. Mech. *13*, dmm043513. https://doi.org/10.1242/dmm.043513.

45. Brooks, B.R., Miller, R.G., Swash, M., and Munsat, T.L. (2000). El Escorial revisited: revised criteria for the diagnosis of amyotrophic lateral sclerosis. Amyotroph. Lateral Scler. Other Motor Neuron Disord. *1*, 293–299. https://doi.org/10.1080/146608200300079536.

46. Cedarbaum, J.M., Stambler, N., Malta, E., Fuller, C., Hilt, D., Thurmond, B., and Nakanishi, A. (1999). The ALSFRS-R: a revised ALS functional rating scale that incorporates assessments of respiratory function. BDNF ALS Study Group (Phase III). J. Neurol. Sci. *169*, 13–21. https://doi.org/10.1016/s0022-510x(99)00210-5.

47. Dubbioso, R., Spisto, M., Verde, L., Iuzzolino, V.V., Senerchia, G., Salvatore, E., De Pietro, G., De Falco, I., and Sannino, G. (2024). Voice signals database of ALS patients with different dysarthria severity and healthy controls. Sci. Data *11*, 800. https://doi.org/10.1038/s41597-024-03597-2.

48. Kimura, F., Fujimura, C., Ishida, S., Nakajima, H., Furutama, D., Uehara, H., Shinoda, K., Sugino, M., and Hanafusa, T. (2006). Progression rate of ALSFRS-R at time of diagnosis predicts survival time in ALS. Neurology *66*, 265–267. https://doi.org/10.1212/01.wnl.0000194316.91908.8a.

YMTHE, Volume *34*

Supplemental Information

siRNA for REST ameliorates symptoms in ALS mice and serum REST predicts disease prognosis and survival in ALS patients

Natascia Guida, Valeria Valsecchi, Serenella Anzilotti, Raffaele Dubbioso, Ornella Cuomo, Silvia Ruggiero, Gianmaria Senerchia, Valentina Virginia Iuzzolino, Xhesika Kolici, Nunzia De Iesu, Giuseppe Pignataro, Lucio Annunziato, and Luigi Formisano

Supplemental Information

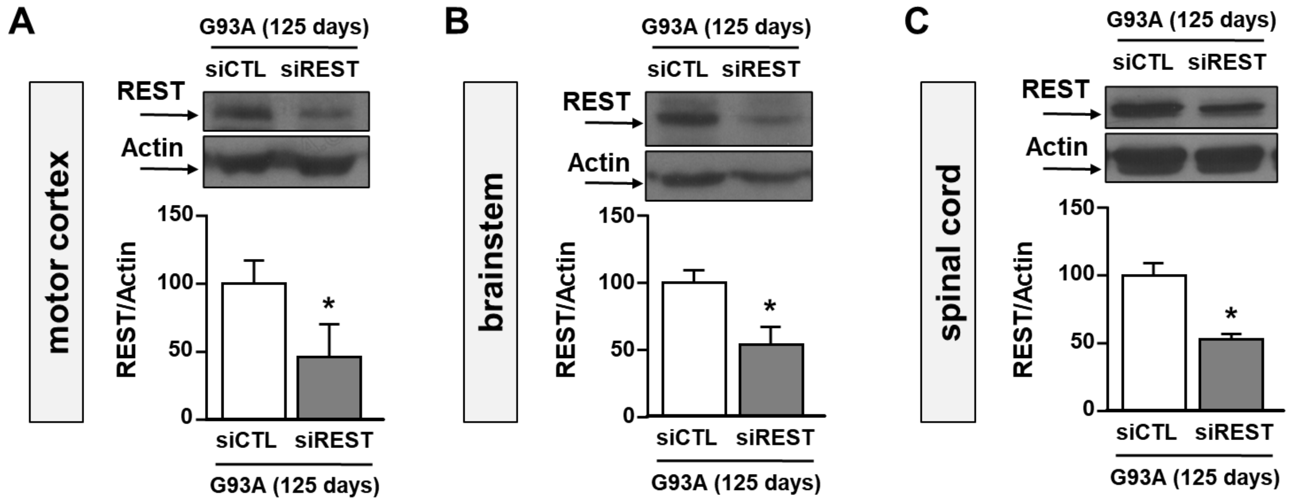

Figure S1

Effect of siREST icv injections on REST protein expression in motor cortex, brainstem and spinal cords of SOD1-G93A mice at 125 days of age. (A-C) Representative western blot and quantification of REST protein levels in motor cortex, brainstem and spinal cords of WT mice treated with siCTL or siREST and sacrificed at 125 days of age after 4 weeks of treatment (N=3). *P < 0.05 vs siCTL by student's t test.

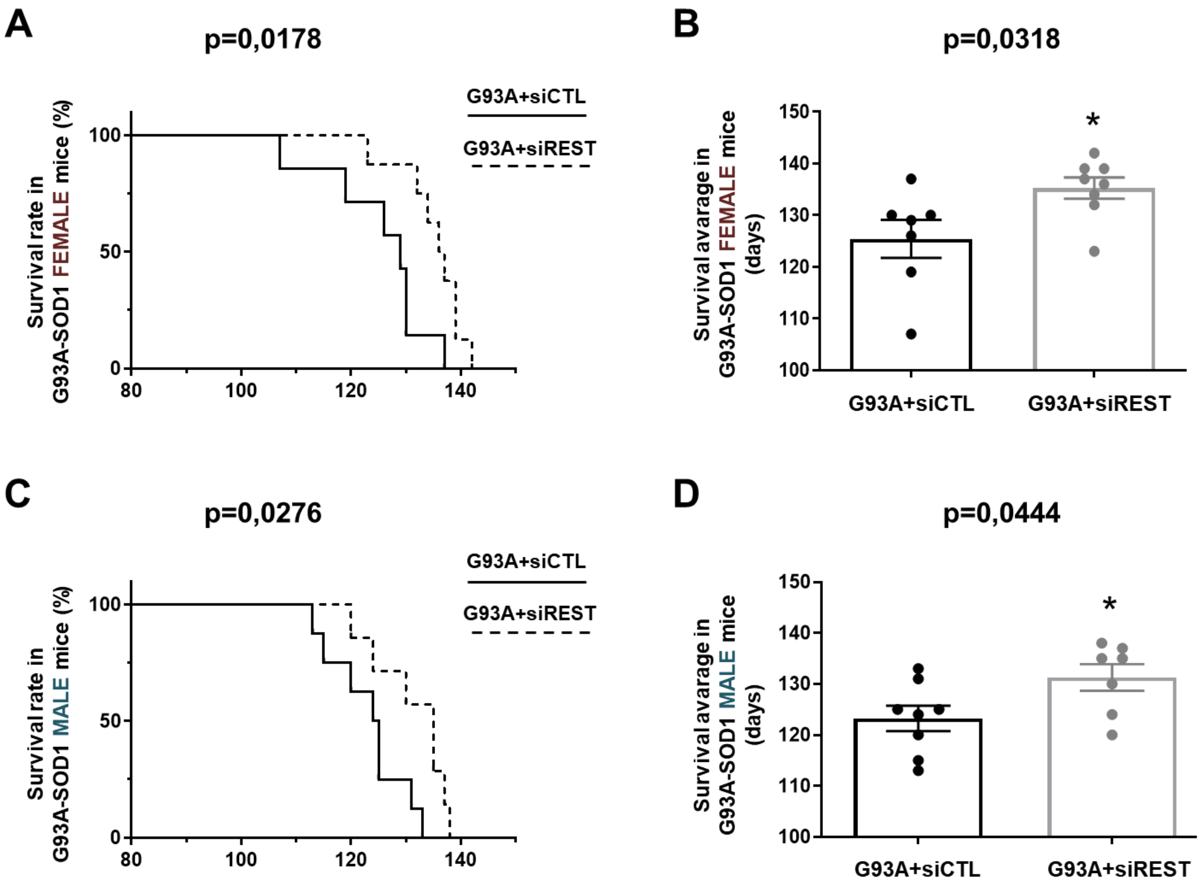

Figure S2

Sex-stratified survival analysis in G93A mice treated with siCTL or siREST. Kaplan-Meier survival analysis, expressed as a percentage (A, C) or in days (B, D) of G93A+siCTL (continuous line and black dots) and G93A+siREST (dashed line and grey dots) female mice (A, B) and male mice (C, D). *P < 0.05 by log-rank statistical test for (A, C) and *P < 0.05 by student's t test for (B, D).

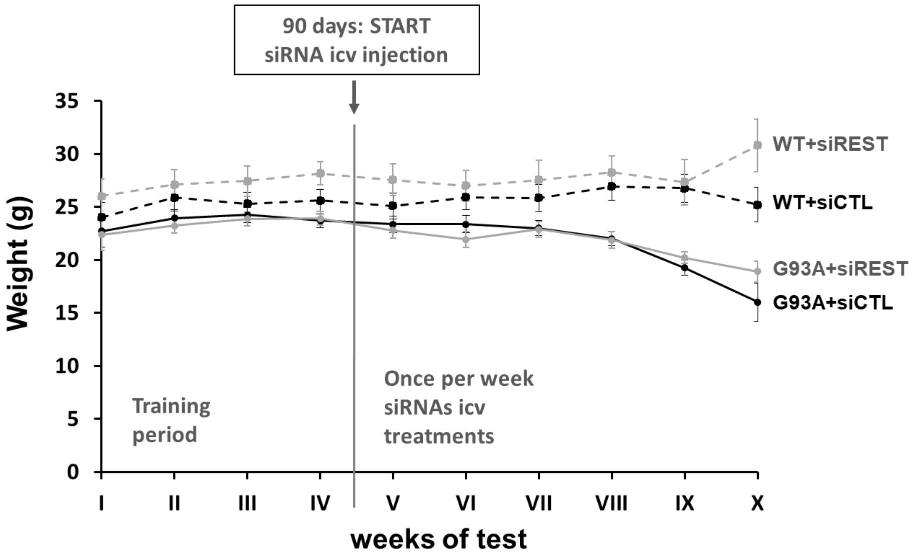

Figure S3

Effect of siREST treatment on body weight of ALS mice. Growth curves of: WT+siCTL (black dashed line; N=13) WT+siREST (gray dashed line; N= 14), G93A+siCTL (black continuous line; N= 28), G93A+siREST (gray continuous line; N= 34) mice. No significant differences by two-way ANOVA analysis, followed by Bonferroni post hoc test.

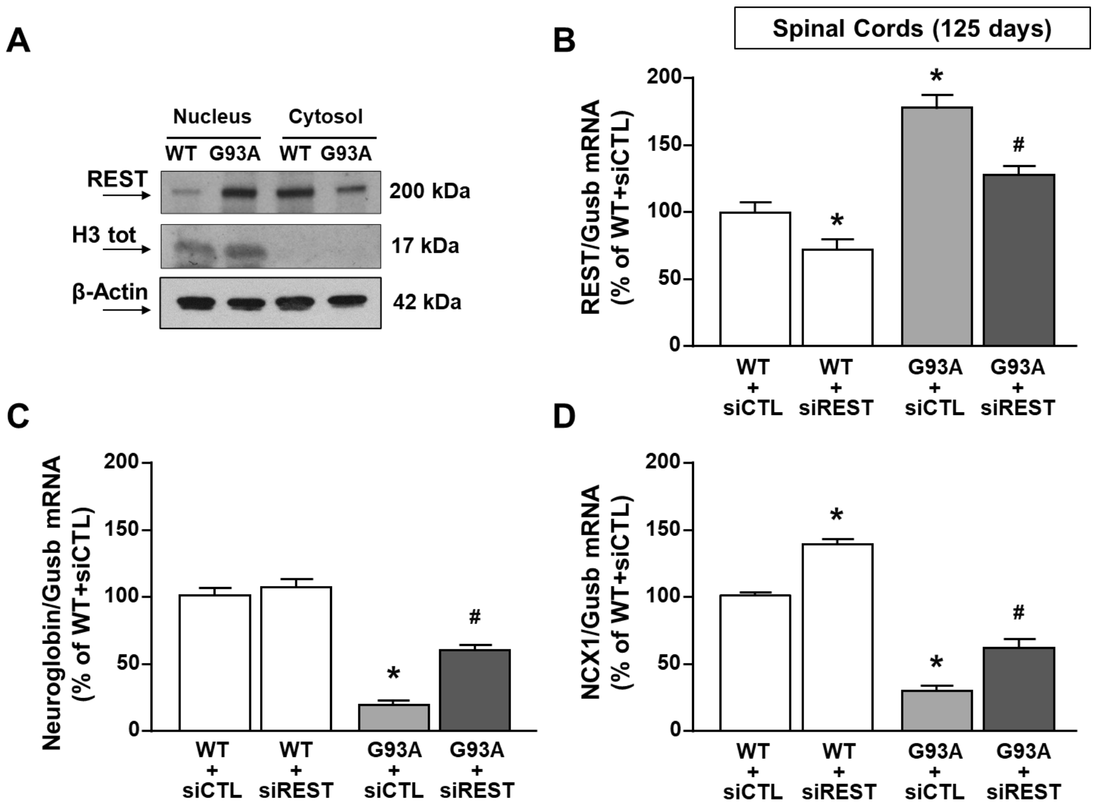

Figure S4

Nuclear and cytosolic REST protein expression and effect of siREST treatment on target genes expression in spinal cords of WT and G93A mice at 125 days of age. (A) Representative western blot of REST protein levels in nuclear and cytoplasmic differentiated lysates from spinal cords of WT and G93A mice at 125 days. Anti-histone H3 (H3) was used to verify the purity of the cytoplasmic and nuclear fractions (N = 3). (B-D) RT-PCR analysis showing REST, Neuroglobin and NCX1 gene expression in spinal cords from: WT+siCTL, WT+siREST, G93A+siCTL, G93A+siREST mice (N=5). *$P < 0.05$ vs WT+siCTL; $^{\#}P < 0.05$ vs G93A+siCTL by one-way ANOVA analysis, followed by Bonferroni post hoc test.
